# The crosstalk between Target of Rapamycin (TOR) and Jasmonic Acid (JA) signaling existing in *Arabidopsis* and cotton

**DOI:** 10.1038/srep45830

**Published:** 2017-04-04

**Authors:** Yun Song, Ge Zhao, Xueyan Zhang, Linxuan Li, Fangjie Xiong, Fengping Zhuo, Chaojun Zhang, Zuoren Yang, Raju Datla, Maozhi Ren, Fuguang Li

**Affiliations:** 1 State Key Laboratory of Cotton Biology, Cotton Research Institute, Chinese Academy of Agricultural Sciences, Anyang, Henan Province, China; 2 School of Life Sciences, Chongqing University, Chongqing, China; 3 National Research Council of Canada, Saskatoon, Canada

## Abstract

Target of rapamycin (TOR) acts as an important regulator of cell growth, development and stress responses in most examined diploid eukaryotes. However, little is known about TOR in tetraploid species such as cotton. Here, we show that TORC1-S6K-RPS6, the major signaling components, are conserved and further expanded in cotton genome. Though the cotton seedlings are insensitive to rapamycin, AZD8055, the second-generation inhibitor of TOR, can significantly suppress the growth in cotton. Global transcriptome analysis revealed that genes associated with jasmonic acid (JA) biosynthesis and transduction were significantly altered in AZD8055 treated cotton seedlings, suggesting the potential crosstalk between TOR and JA signaling. Pharmacological and genetic approaches have been employed to get further insights into the molecular mechanism of the crosstalk between TOR and JA. Combination of AZD8055 with methyl jasmonate can synergistically inhibit cotton growth, and additionally JA levels were significantly increased when cotton seedlings were subjected to AZD8055. JA biosynthetic and signaling mutants including *jar1, coi1-2* and *myc2-2* displayed TOR inhibitor-resistant phenotypes, whereas *COI1* overexpression transgenic lines and *jaz10* exhibited sensitivity to AZD8055. Consistently, cotton *JAZ* can partially rescue TOR-suppressed phenotypes in *Arabidopsis*. These evidences revealed that the crosstalk between TOR and JA pathway operates in cotton and *Arabidopsis*.

Target of rapamycin (TOR) is a serine/threonine protein kinase and is evolutionally conserved from the last eukaryotic common ancestor (LECA) to humans^1^,^2^,^3^,^4^. TOR proteins in these diverse species consist of several highly conserved signature domains including HEAT (Huntingtin, elongation factor 3, regulatory subunit A of PP2A, TOR1) repeats, FAT (the FRAP, ATM, and TRRAP) domain, FRB (FKBP12-rapamycin-binding) domain, kinase domain and FATC (Carboxy-terminal FAT) domain^1^,^4^. In yeasts and mammals, TOR is present in two functionally distinct complexes named TORC1 (TOR complex 1) and TORC2 (TOR complex 2). TORC1 contains TOR, regulatory-associated protein of mTOR (RAPTOR) and lethal with SEC13 protein 8 (LST8), and is rapamycin sensitive. TORC2, which is rapamycin insensitive, includes TOR, LST8, rapamycin-insensitive companion of mTOR (RICTOR) and stress activated map kinase-interacting protein 1 (SIN1)^3^. Rapamycin (RAP) is an antibiotic produced by *Streptomyces hygroscopicus*, and has aided a wealth of studies in animals and yeasts^1^,^3^. However, TOR is insensitive to RAP in majority of land plants for instance *Arabidopsis* and *Vicia faba*, likely due to the altered structural aspects of FKBP12 (FK506 binding protein of 12 kDa) in the formation of functional TOR-rapamycin-FKBP12 ternary complex^5^,^6^.

Compared to great progress made in the understanding of TOR signaling in animals and yeasts, relatively less is known about this ancient and important regulatory system in higher plants. To explore TOR functions using RAP in higher plants, yeast *FKBP12*-based *Arabidopsis* transgenic plants (BP12-2) were produced, which confer conditional sensitivity to rapamycin^7^. Recent studies have also revealed that the ATP-competitive TOR specific inhibitors, which were named after active-site TOR inhibitors (asTORis) including AZD8055 (AZD), Torin1 and Torin2, can also effectively inhibit TOR activity and retard plant growth^8^,^9^,^10^. These inhibitors are effective against both TORC1 and TORC2 complexes and have broader kinase-dependent inhibition on TOR than the widely used rapamycin. Since TOR has many upstream signaling inputs and downstream signaling outputs in eukaryotic organisms, and different signaling pathways associated with these confer different functions, both rapamycin and asTORis could potentially be applied for advancing the underpinning mechanisms in plants. A recent transcriptome analysis of *Arabidopsis* seedlings exposed to AZD showed that TOR regulated photosynthesis and phytohormone signaling pathways including jasmonic acid (JA) signaling pathway^11^. Although these results implied the potential crosstalk between TOR and JA, direct experimental evidence supporting this interaction remains largely elusive.

The phytohormone JA regulates a broad spectrum of biological processes, including cell growth and development, as well as defense responses to biotic and abiotic stresses^12^,^13^,^14^. In the past decades, remarkable progress has been made in understanding of JA biosynthesis and its signaling transduction^13^,^14^,^15^. The bioactive jasmonoyl-isoleucine (JA-Ile), whose formation is catalyzed by JAR1 (Jasmonoyl isoleucine conjugate synthase1), is perceived by the SCF^COI1^ (Skp1/Cullin/F-box) complex that contains CORONATINE-INSENSITIVE1 (COI1) F-box protein and the transcript repressor JASMONATE-ZIM DOMAIN (JAZ) protein^16^,^17^,^18^,^19^,^20^,^21^,^22^. After perception, JAZ proteins were degraded through the 26S proteasome. The bHLH (basic helix-loop-helix) transcription factors MYC2 (Myc transcription factor 2) and MYC3 (Myc transcription factor 3) are the most well characterized regulatory components targeted by JAZ^23^.

Cotton (*Gossypium hirsutum*) provides the major resource of textile fiber worldwide. Environmental stresses are major threats to cotton crop yields. To deal with environmental stresses, crop plants like cotton frequently encounter yield penalties resulting from effects on growth and development. Relevant to these, TOR is involved in regulating plant growth and JA plays a key role in response to environmental stresses^1^,^24^. Dissecting the underlying genetic components and the mechanistic insights linking TOR and JA in plants may provide alternate approaches to develop tolerance against environmental stress and thereby reduce production losses. In this study, we addressed this issue and established that TOR signaling had a significant influence on JA biosynthesis and the associated signal transduction pathways in cotton and *Arabidopsis*. Our results provide new evidences showing a connection between TOR and JA signaling pathways and demonstrate that TOR has a negatively effect on JA signaling pathway.

## Results

### TOR signaling pathway in *G. hirsutum*

**Figure 1:**
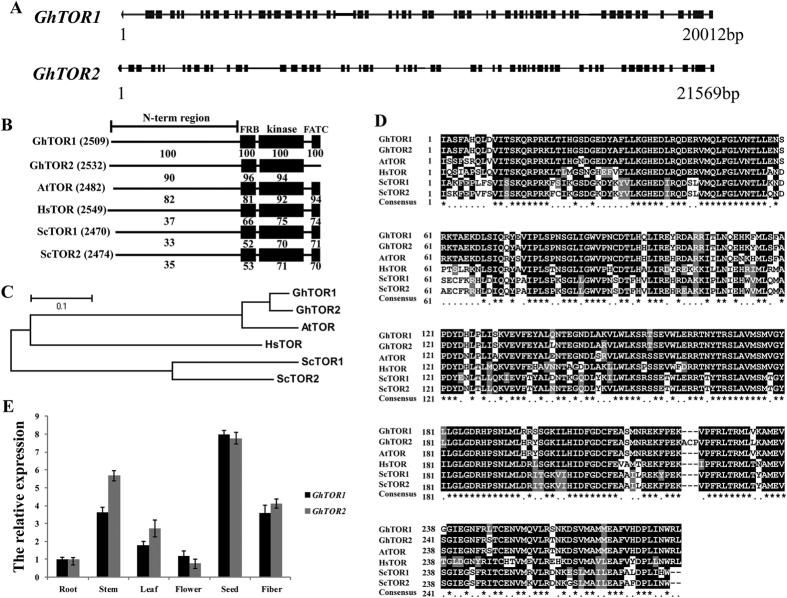
Structures, sequences, and expression analysis of *GhTOR* genes. (**A**) Structure of *GhTOR* genes. Lines represent introns and the solid black rectangles signify exons. (**B**) Comparison of cotton TOR protein sequences with that from other organisms. Each value indicates the percentage of identity with the corresponding domain sequences of GhTOR1. The number in brackets represents the number of amino acids. (**C**) Phylogenetic relationship between the cotton TOR proteins and homologs from other organisms. The phylogenetic tree was generated with MEGA4.0 using the neighbor-joining methods. (**D**) Comparison of amino acid sequences of the kinase domains of cotton TOR proteins with that from other representative organisms. (**E**) Expression levels of two *GhTOR* genes in various cotton tissues. Error bars represent ± SD for three independent experiments. Gh, *Gossypium hirsutum*; At, *Arabidopsis thaliana*; Hs, *Homo sapiens*; Sc, *Saccharomyces cerevisiae*.

The availability of full genome sequences across large number of eukaryotes has significantly expanded our knowledge of TOR signaling pathway^2^. Recently sequenced *Gossypium hirsutum* genome has provided us an opportunity to identify these evolutionary conserved TOR signaling pathway components in tetraploid species cotton^25^. We found the putative homologous gene sequences encoding the key proteins of TORC1 complex including TOR, RAPTOR, and LST8; however, no putative homologs of TORC2-specific proteins, such as RICTOR and SIN1, were present in cotton genome (Table 1 and Supplementary Table 1). Further analysis revealed that the homologs of *HsTOR* were presented at two genetic loci in *G.hirsutum* genome (Fig. 1A). The full-length gene sequence of *GhTOR1 (Target of Rapamycin 1*) spans 20,012 bp and contains 58 exons, and *GhTOR2 (Target of Rapamycin 2*) has 21,569 bp with the same number of exons as observed in *GhTOR1. Gh*TOR1 protein sequences showed similar organization of domains as *Arabidopsis*, humans and yeasts, whereas the FATC domain was not detected in *Gh*TOR2 protein (Fig. 1B). Phylogenetic analysis (Fig. 1C) and kinase domain sequence alignment with that from other representative organisms (Fig. 1D) indicated that *Gh*TOR1 and *Gh*TOR2 were evolutionarily conserved. Moreover, QRT-PCR (Quantitative real time PCR) analysis revealed that the expression of these two *GhTOR* genes can be detected with similar profiles in all cotton tissues (Fig. 1E). It should be noted that besides the two *TOR* homologs, four putative *RAPTOR* homologs were found, but only one copy of *LST8* and *FKBP12* can be detected in cotton genome (Supplementary Table 1). These observations indicate that *TOR* and *RAPTOR* have been duplicated during the evolution of cotton genome from diploid to heterotetraploid whereas one copy of *LST8* and *FKBP12* was lost during this process, indicating that TOR and RAPTOR may play more crucial parts in the evolutionary history and life strategies of cotton (Supplementary Table 1).

IRS (Insulin receptor substrate)-PI3K (Phosphoinositide 3-kinase)-PDK (3′-phosphoinositide-dependent protein kinase)-Akt-TSC1 (Tuberous sclerosis 1)/TSC2 (Tuberous sclerosis 2) signaling cascade is a crucial upstream regulatory input of TOR in humans^1^,^4^. Except PDK, no homologs of other components were present in cotton (Table 1). Since LKB (Loss of liver kinase B1)-AMPK (AMP-activated protein kinase) is another upstream signaling component of TORC1^26^,^27^, we investigated the corresponding putative homologs of LKB and AMPK. Two copies of *LKB* and three copies of *AMPK* were identified and they all displayed high similarities with the corresponding *Arabidopsis* homologs (Supplementary Table 1), indicating that LKB-AMPK, the major energy sensor for ATP/AMP, is a shared signaling factor upstream of TOR in *Arabidopsis* and cotton. Furthermore, our analysis also identified the downstream targets of TOR that include the homologs of S6K (S6 kinase), RPS6 (Ribosomal protein S6), α4, E2F3 (E2F transcription factor 3), ATPBD3 (Repressor of LRR-extension 1), and TCTP (Translationally controlled tumor protein); however, the homologs of VPS34 (Vacuolar protein sorting 34), 4EBP1 (Translation initiation factor 4E-binding protein 1), and SGK1 (Serum/glucocorticoid-regulated kinase 1) were not detected (Table 1 and Supplementary Table 1). It should be noted that additional copies of *S6K* (6 copies) and *RPS6* (ten copies) have been found, suggesting the expansion of these genes in cotton genome compared to the other TOR components like α4 (1 copy), E2F3 (2 copies), ATPBD3 (1 copy) and TCTP (2 copies) in cotton (Supplementary Table 1). The extensive expansion of *S6K-like* and *RPS6-like* genes along with the duplications of *TOR* and *RAPTOR* suggests that TOR/RAPTOR-S6K-RPS6 signaling cascade may act as the mainstay of highly conserved TOR signaling pathways in controlling cell growth and proliferation in cotton.

### AZD but not RAP can efficiently inhibit the growth of cotton seedlings in a dose dependent manner

RAP is a well-known TOR inhibitor and is extensively employed in deciphering plant TOR functions^3^,^7^. RAP depends on the presence of functional FKBP12 to realize its inhibitory effect on TOR protein^7^,^28^. We analyzed the sequences of *GhFKBP12* in the cotton genome (http://cgp.genomics.org.cn). Only a single copy of *FKBP12* (CotAD_57498) was present and was evolutionary conserved among species (Supplemental Fig. 1A). Besides, we performed real-time PCR analyses of *GhFKBP12* and found that *GhFKBP12* had a similar expression pattern to that of *GhTOR* (Supplemental Fig. 1B). Next, to get functional insights into this gene, cotton seedlings and *GhFKBP12* overexpression transgenic *Arabidopsis* lines were exposed to RAP treatment (Supplemental Fig. 1C–H). They all displayed insensitivity to rapamycin, and this observation was consistent with that reported in *Arabidopsis*^6^,^28^. Sequence analysis of GhFKBP12 and homologs of other organisms indicated that alternations in some residues of this protein in cotton likely prevented the formation of a TOR-rapamycin-FKBP12 ternary complex, contributing to the observed RAP insensitivity in cotton (Supplemental Fig. 1A)^6^.

**Figure 2:**
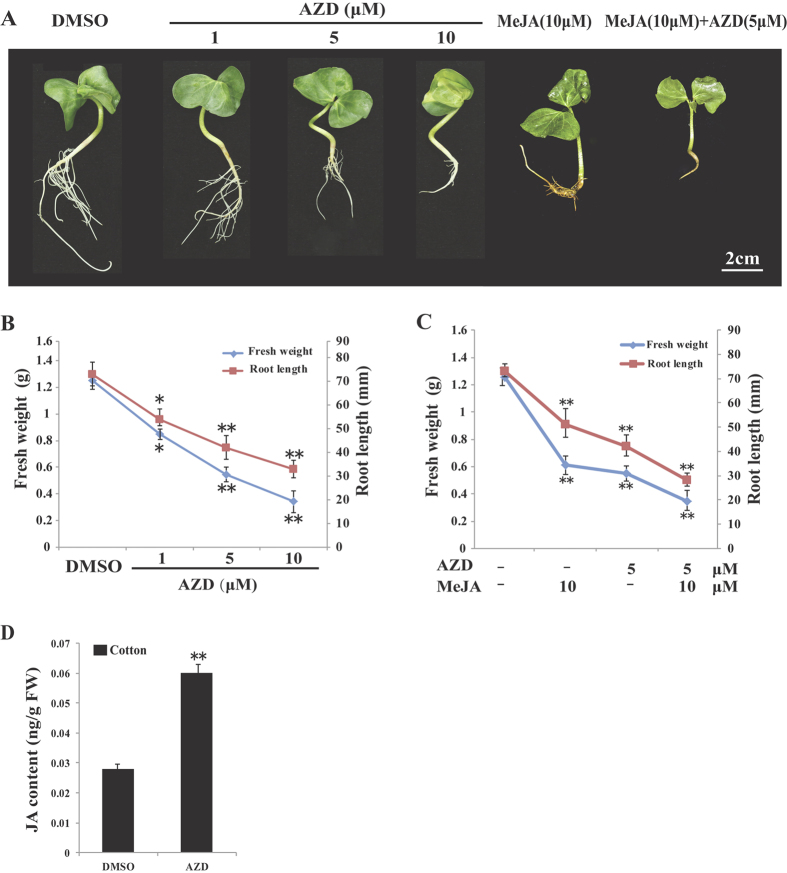
Effect of MeJA and AZD on cotton seedling growth. (**A**) Cotton seedlings with the treatment of DMSO, different concentrations of AZD, MeJA and combination of MeJA and AZD for ten days. Scale bar = 2 cm. (**B**) Fresh weight and root length of cotton seedlings grown on 1/2 MS medium containing DMSO and different concentrations of AZD. (**C**) Fresh weight and root length of cotton seedlings grown on 1/2 MS medium containing DMSO, AZD, MeJA and the combination of AZD and MeJA. Each graph in (**B**) and (**C**) represents the average of 10 seedlings that were conducted in triplicate. Error bars in (**B**) and (**C**) represent ± SD (n = 3). Asterisks indicate significant differences in root length or fresh weight between DMSO and indicated treatments at *p < 0.05 and **p < 0.01 (Student’s t-test). (**D**) JA contents in cotton seedlings exposed to DMSO and 5 μM AZD. Error bars represent ± SD (n = 3). Each independent experiment contains 10 plants per treatment. JA is quantified once for the pool of 10 plants. Asterisks indicate significant differences in JA content between DMSO and AZD treatment at **p < 0.01 (Student’s t-test). MS: Murashige & Skoog.

Given the RAP insensitivity of cotton seedlings, the second generation TOR inhibitor AZD was explored to dissect TOR pathway functions in cotton (Fig. 2A,B). We performed the established functional assay to test the effects of different AZD concentrations on cotton seedling growth. We found that AZD can induce growth retardation of cotton seedlings, and these effects were dose-dependent (as indicated by root length and fresh weight). This suggests that AZD based approach can be used to probe TOR signaling in cotton similar to other plants^8^,^9^.

### Transcriptome analysis of TOR inhibited cotton

**Figure 3:**
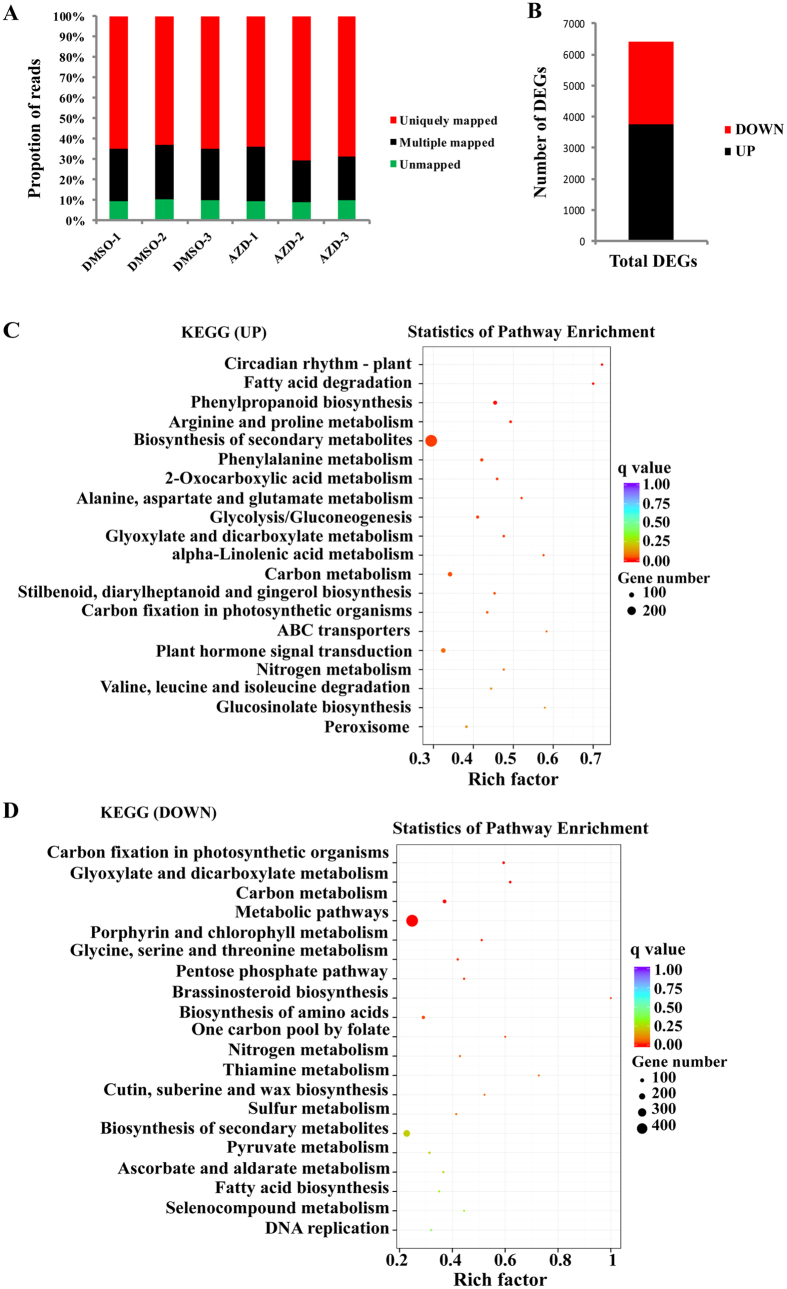
RNA-Seq analysis of the *G.hirsutum* seedlings exposed to AZD. (**A**) Proportions of clean reads of unmapped, mapped to unique genes and mapped to multiple genes, which were plotted by three replicates of DMSO and three replicates of AZD on the horizontal axis. (**B**) Number of up-regulated and down-regulated DEGs. (**C**) Significantly enriched KEGG pathway for up-regulated DEGs. (**D**) Significantly enriched KEGG pathway for down-regulated DEGs. The pathways in (**B**) and (**C**) were ranked by their significance. DEGs: Differentially Expressed Genes. KEGG: Kyoto Encyclopedia of Genes and Genomes.

To further dissect TOR functions in cotton, cotton seedlings treated with DMSO (as a control) and AZD were used for RNA-seq analysis. After trimming for quality, 87.77 and 96.96 million RNA-seq clean reads were obtained under the treatment of DMSO and AZD (representing the average of three replicates), respectively (Supplementary Table 2). The corresponding raw datasets and processed files of the RNA-seq data have been deposited in NCBI’s Sequence Read Archive (SRA) under accession number SRX2568809. Approximately 90% of the reads in each of the three replicates were mapped to the cotton genome (Fig. 3A). A total of 6419 differentially expressed genes (DEGs) were found between DMSO and AZD treatment; among these 3767 were up-regulated and 2652 were down-regulated (Fig. 3B). KEGG (Kyoto encyclopedia of genes and genomes) pathway analysis revealed that the up-regulated genes were mainly enriched for several processes including circadian rhythm, fatty acid biosynthesis and phenylpropanoid biosynthesis (Fig. 3C). Besides these, the carbon fixation associated with photosynthesis showed the highly enriched category among the down-regulated genes (Fig. 3D). The plant hormones signal transduction pathway and carbon fixation pathway are consistent with previously reported observations in TOR inhibited *Arabidopsis*^11^. Interestingly, the differentially expressed genes also include some key JA biosynthetic and transduction associated genes, suggesting potential interaction between TOR and JA (Table 2). Furthermore, we also found the altered expression profiles of auxin and abscisic acid (ABA) related genes under TOR inhibition in cotton; these observations are highly consistent with similar treatments in *Arabidopsis*^11^ (Supplementary Table 3). As the crosstalk between TOR and ABA or auxin signaling pathways have already been previously investigated^9^,^29^,^30^, we focused on JA signaling pathway for further investigation. The expression levels of some randomly-selected genes were verified using QRT-PCR, and the results were consistent with the RNA-Seq analysis (Supplementary Figure 2).

### AZD can significantly enhance MeJA-induced growth inhibition on cotton seedlings

Next, we examined whether the potential crosstalk between TOR and JA exists in cotton by applying MeJA (Methyl jasmonate), AZD, and a pairwise combination of MeJA + AZD to cotton seedlings (Fig. 2A,C). The roots of MeJA-treated cotton seedlings turned brown in color, most likely due to the accumulation of polyphenols and anthocyanin. Furthermore, growth inhibition inflicted by the combination of AZD + MeJA in cotton seedlings was more pronounced than the treatments with AZD or MeJA alone. Furthermore, JA level increase induced by TOR inhibition was also observed in cotton seedlings (Fig. 2D). These results indicated that the interplay between TOR and JA may exist in cotton.

### MeJA and TOR inhibitors synergistically inhibit plant growth

**Figure 4:**
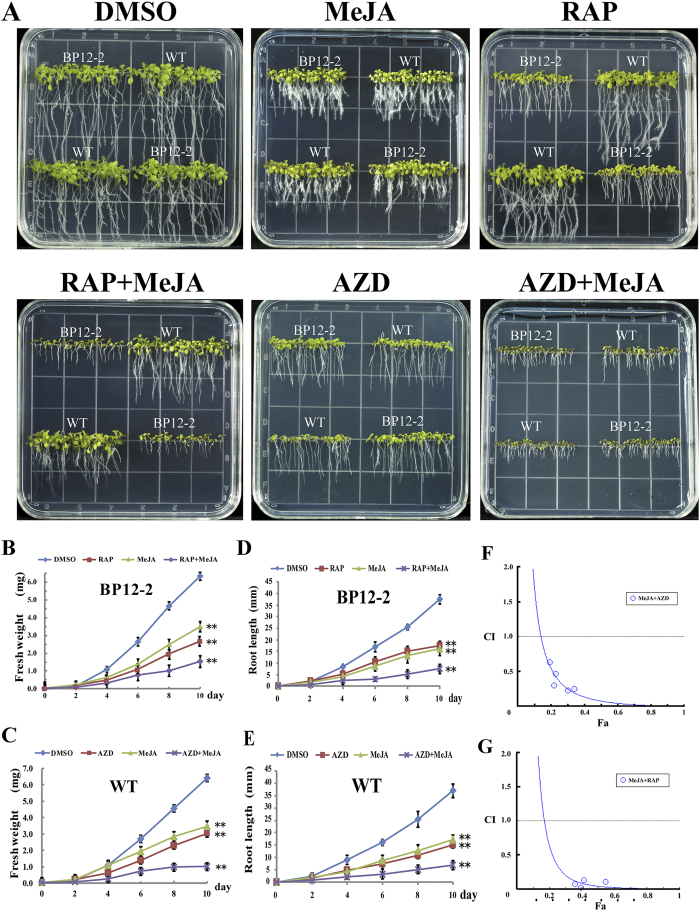
TOR inhibitors and MeJA synergistically inhibit the growth of *Arabidopsis* seedlings. (**A**) Phenotypes of 10-day-old WT and BP12-2 seedlings exposed to DMSO, 10 μM MeJA, 0.35 μM RAP, 1 μM AZD and the combination of MeJA + RAP or MeJA + AZD. Fresh weight (**B**) and root length (**D**) of BP12-2 seedlings treated with DMSO, 0.35 μM RAP, 10 μM MeJA and the combination of MeJA + RAP. Fresh weight (**C**) and root length (**E**) of WT seedlings treated with DMSO, 1 μM AZD, 10 μM MeJA and the combination of MeJA + AZD. Each graph in (**B**–**E**) represents the average of 10 seedlings that were conducted in triplicate. Error bars represent ± SD (n = 3). Asterisks indicate significant differences in root length or fresh weight between DMSO and indicated treatments at **p < 0.01 (Student’s t-test). (**F**–**G**) Fa-CI curve shows synergism (CI < 1) between MeJA and TOR inhibitors.

Due to the lack of mutant collections and difficulties in genetic transformation in cotton, we used the model plant *Arabidopsis* to study the potential interactions between TOR and JA. RAP selectively inhibits TORC1-S6K signaling while AZD is a pan-TOR inhibitor which can directly interact with kinase domain of TOR by competing with ATP. These different properties of RAP and AZD may provide alternative and complementary approaches to investigate the crosstalk between TOR and JA in *Arabidopsis*. WT and BP12-2^7^ were exposed to MeJA, TOR inhibitors (AZD or RAP) and the pairwise combination of RAP + MeJA or AZD + MeJA (Fig. 4A–E). As expected, WT and BP12-2 seedlings grown on 1/2 MS (Murashige & Skoog) medium supplemented with DMSO, MeJA, RAP and AZD showed phenotypes similar to previous observations^7^,^8^,^31^. However, the combinatorial treatment using MeJA + RAP resulted in BP12-2 but not WT seedlings undergoing enhanced inhibition (regarding fresh weight and root length) (Fig. 4A,B and D). Besides, plants treated with AZD + MeJA showed more pronounced inhibition than with AZD or MeJA alone (Fig. 4A,C and E). These results indicated that MeJA and TOR inhibitors may synergistically inhibit plant growth.

To quantitatively measure the synergism generated by the interaction between TOR inhibitors and MeJA, CI (Combination index) values were used in this study. First, to further examine whether the synergism can be generated by the combination of TOR inhibitors (RAP or AZD) with MeJA, we investigated the GI50 (50% growth inhibitory dose) values of each drug alone and pairwise combinations in BP12-2 seedlings (Supplementary Table 4). The GI50 values of MeJA, RAP and AZD were 10, 0.35 and 1 μM, respectively. However, their GI50 values were significantly reduced when BP12-2 were subjected to MeJA + RAP or MeJA + AZD treatments, implying that the potential synergistic effects can be generated by combining MeJA and TOR inhibitors. Next, computer-simulated Fa (Affected fraction)-CI curves were assessed by using the CompuSyn software (Fig. 4F,G). Synergistic effects (CI < 1) were observed when BP12-2 plants were treated with a combination of MeJA + RAP or MeJA + AZD, showing that MeJA + RAP or MeJA + AZD interaction had synergism. These results confirmed that MeJA and TOR inhibitors (AZD or RAP) could synergistically inhibit plant growth.

### TOR negatively influences JA biosynthesis and plant response to JA

**Figure 5:**
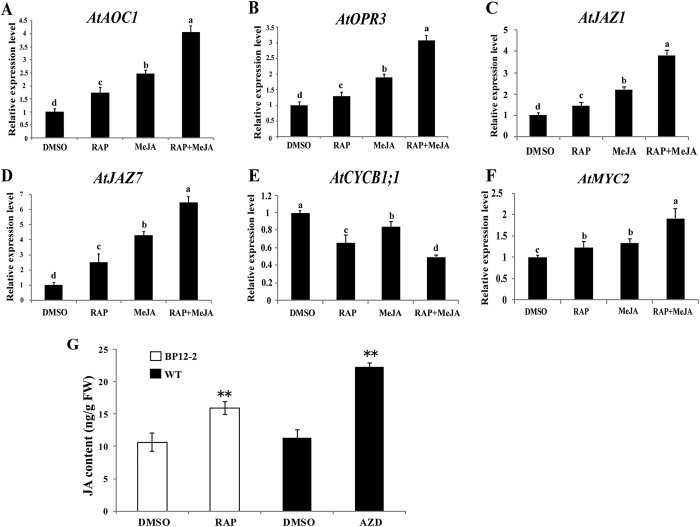
TOR inhibition influences JA biosynthesis and response in plants. 6-day-old BP12-2 seedlings were treated with DMSO, 0.35 μM RAP, 10 μM MeJA and the combination of MeJA + RAP for 24 h. Relative expression levels of JA biosynthetic genes *AtAOC1* (**A**) and *AtOPR3* (**B**), and JA responsive genes *AtJAZ1* (**C**), *AtJAZ7* (**D**), *AtCYCB1;1* (**E**) and *AtMYC2* (**F**) were examined. The expression levels of these genes in DMSO were set to 1. (**G**) JA contents in BP12-2 and WT seedlings exposed to TOR inhibitors. Each independent experiment contains 10 plants per treatment. JA is quantified once for the pool of 10 plants. Error bars represent ± SD (n = 3). Three biological replicates were analyzed with similar results. Asterisks (**p < 0.01) and different letters indicate significant differences from the control.

To further dissect the crosstalk between TOR and JA signaling pathway, we examined the effect of TOR inhibition on the mRNA levels of the major JA biosynthetic and responsive genes. The transcription levels of six genes including the major JA biosynthetic genes *AOC1 (Allene oxide cyclase1*) and *OPR3 (OPDA reductase3)*^32^,^33^ that have a close relationship with JA content in plants, repressors of JA signaling pathway *JAZ1* and *JAZ7*^17^,^18^,^19^ whose transcript levels showed significant changes in the RNA-seq data of TOR-inhibited *Arabidopsis* seedlings^11^; cell cycle related gene *CYCB1;1 (Cyclin-dependent protein kinase1;1)*^34^ which is greatly affected by JA signaling pathway; and the bHLH transcription factor *MYC2*^23^ which functions as a regulator of JA-responsive gene expression were analyzed (Fig. 5A–F and Supplementary Figure 3B). In both BP12-2 transgenic lines (Fig. 5A–F) and WT *Arabidopsis* (Supplementary Figure 3B), TOR inhibition and MeJA treatments resulted in a decrease in the relative transcript levels of *CYCB1;1*, but led to an increase in the expression levels of the other five genes. Besides, when plants were treated with the combination of TOR inhibitor and MeJA, relative mRNA levels of these genes were changed in a more dramatic manner than those treated with single drug alone. The CI curves of two selected genes suggested that MeJA and TOR inhibitors had a synergistic effect on the mRNA levels of these genes in BP12-2 transgenic lines (Supplementary Figure 3A).

Since TOR suppression increased the relative transcript levels of JA biosynthetic genes (Table 2) and JA levels (Fig. 2D) in cotton, we speculated that TOR inhibition also influenced the endogenous JA content in *Arabidopsis*. Therefore, we measured the endogenous JA levels in *Arabidopsis* (Fig. 5G). Consistently, our results showed that suppression of TOR resulted in the significant increase in JA production in *Arabidopsis*, which was in agreement with the QRT-PCR data shown above. Taken together, our data suggest that TOR has a negative effect on JA biosynthesis and its response.

### Sensitivity of JA synthesis and signaling mutants to AZD

**Figure 6:**
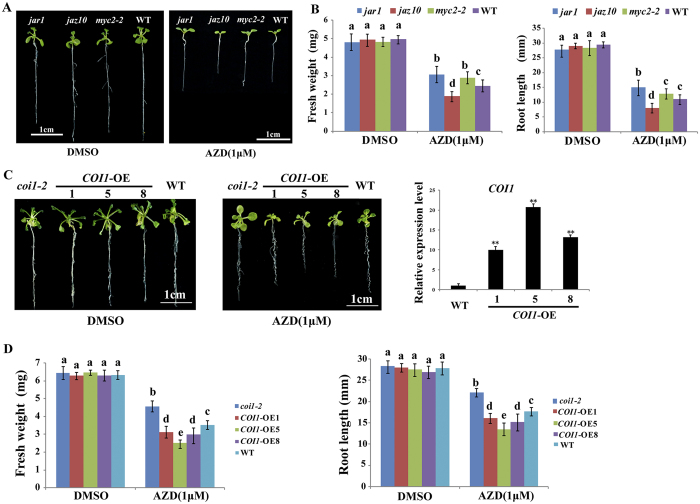
Effect of AZD on JA synthesis and transduction deficient mutants. (**A**) Phenotypes of *jar1, jaz10* and *myc2-2* exposed to DMSO and 1 μM AZD for ten days. Scale bar = 1 cm. (**B**) Fresh weight and root length of *jar1, jaz10* and *myc2-2* seedlings in (**A**). (**C**) The phenotypes of *coi1-2* mutants and *COI1*-overexpression seedlings grown on 1/2 MS medium containing DMSO and 1 μM AZD for 10 d. Scale bar = 1 cm. The relative expression levels of *COI1* in WT and transgenic lines were analyzed by QRT-PCR. Asterisks indicate significant differences in *COI1* expression levels between WT and transgenic lines at **p < 0.01 (Student’s t-test). (**D**) Root length and fresh weight of *coi1-2* mutants and *COI1*-overexpression seedlings in (**C**). Each graph in (**B**) and (**D**) represents the average of 10 seedlings that were conducted in triplicate. Error bars represent ± SD (n = 3). Different letters indicate significant differences from the control. MS: Murashige & Skoog.

Next, we examined whether the mutants *jar1, coi1-2, jaz10* and *myc2-2* deficient in crucial steps of JA synthesis and transduction are affected by AZD treatments (Fig. 6). Interestingly, the results showed that *jar1, coi1-2* and *myc2-2* were insensitive to AZD compared with WT, as indicated by root length and fresh weight data (Fig. 6B and D). In contrast, the transcriptional repressor mutant *jaz10* was sensitive to AZD (Fig. 6A,B). Moreover, the finding that *coi1-2* was insensitive to AZD prompted us to generate *COI1* overexpression transgenic lines (Fig. 6C,D). *COI1*-overexpression transgenic lines were generated by introducing P35S::*COI1*-HA into WT *Arabidopsis*. These *COI1*-overexpression transgenic lines showed AZD-hypersensitivity phenotypes including slower growth, smaller leaf size and shorter root length than WT, which displayed the opposite phenotype observed in *coi1-2* (Fig. 6C). These independent lines of evidences further revealed the crosstalk between TOR and JA signaling pathways at multiple sites and levels.

### *GhTIFY* genes are involved in the crosstalk between TOR and JA

**Figure 7:**
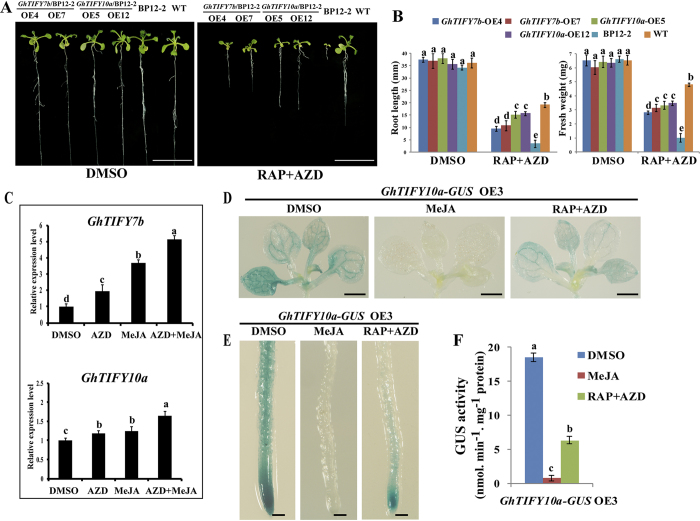
Effect of TOR inhibitors on *GhTIFY* overexpression lines and GhTIFY protein degradation. (**A**) *GhTIFY* overexpression lines were partially insensitive to TOR inhibitors. *GhTIFY7b* and *GhTIFY10a* overexpression transgenic lines were in *Arabidopsis* BP12-2 background and exposed to DMSO and 0.35 μM RAP + 1 μM AZD for ten days. Scale bar = 1 cm. (**B**) Root length and fresh weights of *GhTIFY* overexpression lines in (**A**). Each graph represents the average of 10 seedlings that were conducted in triplicate. (**C**) Analysis of *GhTIFY7b* and *GhTIFY10a* expression levels in cotton seedlings exposed to DMSO, 5 μM AZD, 10 μM MeJA and the combination of AZD + MeJA. The expression levels of these genes in DMSO were set to 1. (**D**,**E**) *35S*-*GhTIFY10a-GUS* OE3 overexpression transgenic lines were treated with DMSO, MeJA (10 μM), or RAP (0.35 μM) + AZD (1 μM) for 48 h. The leaves (**D**) and roots (**E**) were used to perform the GUS staining assays. (**F**) GUS activity quantification of *35S*-*GhTIFY10a-GUS* OE3. In (**C**) and (**F**), Error bars represent ± SD (n = 3). Different letters indicate different significant differences from the control.

The above results indicated that TOR has a negative effect on JA biosynthesis and its signal transduction. JAZs, the subfamily of the TIFY family, are well established transcriptional repressors of JA signaling pathway^17^,^18^,^19^. It is possible that the growth defects caused by TOR suppression can be rescued by *GhJAZ* overexpression. To verify this hypothesis, we next examined the interactions between cotton JAZ/TIFY and TOR by overexpressing cotton *JAZ/TIFY* genes in *Arabidopsis* BP12-2 background. Twenty-one putative *TIFY* genes in *G. hirsutum* were cloned and named following the existing nomenclature system used in *Arabidopsis* (Supplementary Table 5). Phylogenetic construction and motif of these genes were shown in Supplementary Figure 4A. Among the thirteen *JAZ* genes, two *JAZ* overexpression transgenic lines *GhTIFY7b (JAZ7)* and *GhTIFY10a (JAZ10*) showed a relative better growth condition under TOR inhibition than other transgenic lines, thus *GhTIFY7b* and *GhTIFY10a* were selected for further analysis (Fig. 7 and Supplementary Figure 4B,C). We found that these transgenic lines were partly insensitive to TOR inhibitors compared with BP12-2, indicating that *GhTIFY* overexpression can partly rescue the growth defects of TOR suppression (Fig. 7A,B). Besides, when cotton seedlings were exposed to TOR inhibitors and MeJA, the relative transcript level changes of these two genes were consistent with the results in *Arabidopsis* (Fig. 7C).

In *Arabidopsis*, JAZ proteins are degraded by 26 S proteasome when plants respond to JA^17^,^18^,^19^. To verify whether MeJA and TOR inhibitors could induce the degradation of GhTIFY proteins, *35S*-*GhTIFY-GUS* transgenic plants (*GhTIFY7b* and *GhTIFY10a*) were generated and exposed to MeJA and TOR inhibitors (Fig. 7D–F and Supplementary Figure 4C). Consistent with the expectation, MeJA treatment induced significant decrease in GUS activity in these transgenic lines. Interestingly, RAP and AZD can mimic MeJA to promote the degradation of GhTIFY-GUS protein in *Arabidopsis*. These observations further confirmed that TOR is involved in the JA signaling in plants.

## Discussion

Most examined heterotrophic organisms contain two distinct TOR complexes: TORC1 and TORC2, and TOR signaling pathway has been well documented in several model organisms^1^,^2^,^3^,^4^. However, little information is known about TOR signaling pathway in cotton. Based on the recently released genome database of cotton (http://cgp.genomics.org.cn/), we identified the putative components of TOR complexes, the upstream and downstream factors of TOR signaling pathway for the first time. Unlike the FATC domain’s essential functions for TOR protein kinase in yeast and animals^35^, in *Arabidopsis*, this domain appears dispensable and no clear phenotype can be observed with its absence^36^. We found that FATC domain only exists in GhTOR1 but not in GhTOR2. The detailed function of this domain in cotton remains to be understood. Moreover, the significant expansion of TORC1/S6K/RPS6 in cotton genome implies that this signaling cascade may be the mainstay of TOR signaling pathway in cotton. These bioinformatic investigations uncovered an expansion and diversification of *TOR* genes and the canonical TOR components in cotton, which might point to new additional functions for TOR signaling.

Rapamycin and AZD were employed to probe TOR signaling in this study. They both target TOR protein, but the modes of action, molecular mechanism, inhibitory spectrum and drug potency of rapamycin are quite different from that of AZD. Rapamycin is the first generation TOR inhibitor and specifically inhibit TORC1-S6K signaling branch, whereas AZD is the second generation of TOR inhibitor and can suppress most outputs of TORC1 and TORC2. Since TOR has many upstream signaling inputs and downstream signaling outputs in eukaryotic organisms and different signaling cascades operated through these have different functions, both selective TOR inhibitor (rapamycin) and pan-TOR inhibitor (AZD) based approaches are useful for defining TOR signaling in plants. Rapamycin/FKBP12/TOR ternary complex provides a conditional and highly inducible selective system to decipher TORC1-S6K-RPS6 signaling. Considering TORC1-S6K-RPS6 is the mainstay of cotton TOR signaling, rapamycin should be a very important tool to dissect TOR signaling if GhFKBP12 is able to bridge the interaction between RAP and TOR. However, our observations suggest that GhFKBP12 is defective to interact with RAP and not effective to produce TOR inhibition. As an alternative, we have successfully employed AZD to probe TOR signaling in cotton; and the *Arabidopsis* BP12-2 line was successfully applied to detect the functions of GhTIFYs in TOR signaling by using RAP. These results demonstrate that RAP and AZD have many overlapping and complementary advantages for TOR signaling research in cotton and *Arabidopsis*.

Cotton RNA-seq analysis was employed to elucidate the function of TOR signaling. The RNA-Seq analysis show that TOR inhibition result in changes in many metabolic processes, which further implies that TOR plays a key role in cotton growth and development. Besides, plant hormone signal transduction, JA-related genes were significantly altered in TOR inhibited cotton seedlings (Fig. 3C and Table 2). In *Arabidopsis*, the expression profiles of representative JA related genes were also altered under TOR suppression by AZD^11^. These results indicate the potential interaction between JA and TOR in cotton. The precise molecular mechanism that mediates their crosstalk remains to be elucidated. In *Arabidopsis*, TOR inhibition can be partially rescued by a mutation in *ABI4 (ABA Insensitive 4)*, which encodes an ABA-regulated AP2 domain transcription factor^9^. In cotton, there were two putative homologs of ABI4 (CotAD_22351 and CotAD_43460) whose protein sequences show a relatively low identity (46% and 47%) with that of *Arabidopsis*. In the DEGs of our cotton RNA-seq data, we didn’t find these two putative homologs, potentially either due to the loss of the evolutionary functional conservation for these genes or they do not represent the true orthologs. Besides, the transcript levels of the other key node genes of ABA signaling pathway were changed in cotton for instance PYL (PYRABACTIN resistance 1-like), ABF (Abscisic acid responsive elements-binding factor) and PP2C (Type 2C protein phosphatases) family protein (Supplementary Table S3). Repression of ABA signaling is a potential mechanism to enhance JA signaling indirectly.

Interestingly, AZD treatment mimics the growth defects generated by MeJA treatment in cotton seedlings. Importantly, the endogenous levels of JA were significantly increased when cotton seedlings were subjected to AZD, along with the transcriptional changes of JA biosynthetic and responsive genes in both cotton and *Arabidopsis*. TOR inhibition not only altered the expression levels of JA-responsive genes, but also influenced the expression of genes involved in JA biosynthesis. These results suggest important functions for broader crosstalk between JA and TOR pathways in cotton. To test this hypothesis, we performed the pharmacological analysis in cotton by combining AZD with MeJA, and stronger inhibitory effects were observed than that of single drug treatment (Fig. 2). Furthermore, using a statistical analysis software^10^,^37^, we found the growth-inhibition and gene-expression effects of these treatments acted synergistically when *Arabidopsis* were subjected to the combined treatment of RAP + MeJA or AZD + MeJA. These independent lines of evidences strongly suggest the possibility of the interaction between JA and TOR signaling. To further confirm these results, using genetic approaches, mutants deficient in crucial steps of JA synthesis and perception including *jar1, coi1-2, jaz10* and *myc2-2* were employed to examine TOR signaling. Our results also show that the loss of function mutant *jar1, coi1-2* and *myc2-2* was insensitive to AZD, whereas *jaz10* and *COI1* overexpression result in a phenotype, which was a complete opposite to that of the other mutants (Fig. 6). The JAZ protein, which belongs to a subfamily of the TIFY family, is a transcriptional repressor of JA^17^,^18^,^19^. When plants respond to JA, JAZ protein is ubiquitinated and subsequently degraded by the 26 S proteasome. Because the understanding of the plant-specific TIFY family in cotton is limited, we identified 21 putative *TIFY* genes in cotton, analyzed their sequences (Supplementary Figure 4A), and investigated their functions in the model plant *Arabidopsis* (Fig. 7). The results from these studies show that overexpression of the *TIFY* genes could partially compensate the growth inhibition caused by TOR inhibitors, and TOR inhibition could also induce the degradation of the TIFY protein. These results suggest that genetic disruptions of JA signaling reduce the effects of TOR inhibition by using the *coi1-2, jar1*, and *myc2-2* mutants and strong expression of putative JAZ repressors from cotton, while potentiating JA signaling through over-expression of *COI1* and using *jaz10* mutant enhances the growth retarding effects of TOR inhibition.

Based on the findings of the present study, we propose a working model for the interaction between TOR and JA signaling pathways in plants (Supplementary Figure 5). Since TOR signaling pathway plays a key role in plant growth and development, suppression of TOR creates a stress for plants. As JA is an important regulator of plant stress response, TOR inhibition triggers plant stress response by promoting JA biosynthesis and activation of JA signaling. When TOR was inhibited, JA biosynthesis renders JAZ degradation. The genetic evidences presented in this study provide crucial insights into the cross-talk between TOR and JA signaling pathways at multiple levels including JAR1, COI1, MYC2 and JAZ.

## Methods

### Materials and growth conditions

*Arabidopsis thaliana* ecotype Columbia-0 (Col-0) was used as the wild-type plants in this study. The transgenic *Arabidopsis* BP12-2 was generated in a previous study^7^. The *Arabidopsis* mutant lines *jar1, coi1-2, myc2-2* and *jaz10* were kindly provided by Dr. Chuanyou Li of the Institute of Genetics and Developmental Biology, Chinese Academy of Sciences. *Arabidopsis* seeds were surface-sterilized with 70% ethanol for 3 min; followed by 10% bleach containing 0.3% Tween-20 for 10 min; and then rinsed by sterile water for three times. The sterilized seeds were suspended in 0.1% sterile agrose and kept at 4 °C for two days, and then germinated on 1/2 MS (Murashige and skoog) medium plates supplemented with reagents as indicated in Figures. All *Arabidopsis* were grown at 22 °C under long-light conditions (16 h light/8 h dark). Wild-type *G. hirsutum* (CCRI24) is used in this study. Cotton seeds were surface-sterilized with 0.1% mercuric chloride for 5 min, rinsed with sterile water. The sterilized seeds were sown in MS medium containing different solutions and 1% agar, and grown in a growth chamber at 25 °C under 16 h light/8 h dark condition. Plant growth showed no difference between MS and MS + DMSO medium, and all drugs were dissolved in DMSO, MS + DMSO medium was set as a control in this study.

### Combination index (CI) value measurement

Combination index (CI) values were used to quantitatively measure the interaction between MeJA and TOR inhibitors^10^. The degree of reagent interaction is based on synergism (CI < 1), additive effect (CI = 1), or antagonism (CI > 1)^37^. 6-day-old BP12-2 seedlings were incubated in 48-well plates containing 1/2 MS liquid medium supplemented with DMSO (as a control), different concentrations of MeJA and RAP, or a combination of MeJA + RAP for 4 d in a growth chamber with a light intensity of 100 μmol/m^2^s at 22 °C. Fresh weight of 6- and 10-day-old seedlings in every well was measured to calculate growth inhibition. Experiments were repeated three times. Percentage of growth value was calculated by using the fresh weight of 6-day-old seedlings (W6), DMSO-treated control plants (C), and drug-treated plants (D) as follows: [(D-W6)/(C-W6)] × 100. The 50% growth inhibitory dose (GI50) and CI values were calculated using the CompuSyn software program^38^. Fa (Affected fraction) represented the fraction of plants’ fresh weight affected by the reagent. The values of Fa indicated a growth inhibition value and were calculated according to the software instructions as follows: [(100-%growth value)/100].

### Genome-wide identification of *G. hirsutum* TOR signaling pathway components, *FKBP12* and *TIFY* family

*Arabidopsis* TOR and FKBP12 protein sequences were used as quests searching for cotton homologs in the cotton genome database (http://cgp.genomics.org.cn/)^25^. Sequences of humans and *Arabidopsis* TOR signaling pathways components were used to identify cotton homologs in the same database. The 21 *G. hirsutum TIFY* genes were identified by aligning the TIFY domain sequences with the cotton genome (http://cgp.genomics.org.cn/). E-value (1e–10) was set to obtain the final TIFY proteins. Phylogenetic tree was constructed by MEGA4.0 using the neighbor-joining method^39^,^40^. Motif analysis was performed using MEME (http://meme.sdsc.edu/meme)^41^.

### Plasmid constructions and generation of transgenic plants

Full-length CDS were amplified by PCR using the TransStar Taq Polymerase Mix kit (TRANSGEN) following the manufacturer’s instructions. The list of primers is shown in Supplementary Table 6. The recombinant plasmids were generated as described previously^7^. The transgenic lines were generated by *Agrobacterium*-mediated transformation using the floral dip method^42^. Transgenic plants seeds were germinated on 1/2 MS medium containing 50 mg/L kanamycin and confirmed by PCR analysis. The T3 homozygous plants were used in the experiments.

### Quantitative real-time PCR

Total RNA was isolated from cotton or *Arabidopsis* seedlings as indicated in Figures using the RNAprep Pure Plant kit (TIANGEN). PrimeScript RT reagent kit (TAKARA) was used to generate first-strand cDNA products. The CFX96 real-time PCR system (BIO-RAD) was used to measure relative transcript expression levels by using an SYBR Premix Ex Taq II (TAKARA) kit. Primers used for QRT-PCR were listed in Supplementary Table 6. Expression levels of target genes were normalized to *AtACTIN2* and *GhHISTONE3* in *Arabidopsis* and cotton, respectively. The relative quantitative method (2^−ΔΔCT^) was used to calculate the quantitative variation^43^. The data were expressed as the mean ± SD of three independent experiments. At least 5–10 plants of every treatment were sampled for each independent biological replicate.

### GUS staining and GUS activity quantification

X-Gluc was used in GUS staining following a previously reported procedure^44^. Images of the representative lines were captured using a stereomicroscope (OLYMPUS MVX10). A MarkerGene™ β-glucuronidase (GUS) reporter gene activity detection kit (Marker Gene Technologies) was used to quantify GUS activity. The Bradford assay was used for total protein quantification^45^. Three biological experiments, each consisting of 30 plants per treatment were measured.

### JA content measurements

Two-week-old *Arabidopsis* or six-day-old cotton seedlings were transferred to 1/2 MS medium containing DMSO or various TOR inhibitors for 48 hours. Each treated *Arabidopsis* or cotton seedling was harvested for measurements of JA contents. JA was extracted following the exact extraction procedure^46^. JA extraction was quantified by GC-MS (Gas Chromatography-Mass Spectrometer) methods as previously reported^47^. Three biological experiments, each consisting of 10 plants per treatment were measured. JA was quantified once for the pool of 10 plants.

### Transcriptome analysis

4-d-old cotton seedlings were transferred into MS medium containing DMSO and 5 μΜ AZD for 24 h, respectively. Total RNA was isolated as described above. Three biological repeats were employed to transcriptome analysis. Sequencing libraries were generated by NEB Next Ultra™ Directional RNA Library Prep Kit for Illumina (New England Biolabs) following the manufacturer’s recommendations. An Illumina Hiseq 4000 platform was used to sequence these libraries, and 100 bp paired-end reads were generated. TopHat2 was used to map these reads to the *G. hirsutum* transcripts with the help of genome annotation^48^,^49^. The mapped reads were assembled by Cufflinks^50^. Differentially expressed genes (DEGs) were identified using Cuffdiff^51^. To investigate the DEGs between the control and treated libraries, fold change of each gene was calculated as the ratio of read counts in the treated libraries to the read counts in the control library followed by transformation of log2. Genes with a P-value ≤ 0.8 and a |log_2_Ratio| value ≥ 1 were identified as significant differentially expressed genes. KOBAS software was used to test the enrichment of DEGs in KEGG pathways^52^,^53^. KEGG enrichment analysis was obtained with a P-value of < 0.05.

### Statistical analysis

SPSS 20.0 software (IBM, Armonk, NY, USA) was used to perform all statistical analysis of data. The data represent the mean ± SD of n = 3 independent experiments. ANOVA analysis and a Tukey-Kramer multiple comparison test was used for data analysis. P < 0.05 was considered as statistically significant.

## Additional Information

**How to cite this article:** Song, Y. *et al*. The crosstalk between Target of Rapamycin (TOR) and Jasmonic Acid (JA) signaling existing in *Arabidopsis* and cotton. *Sci. Rep.* **7**, 45830; doi: 10.1038/srep45830 (2017).

**Publisher's note:** Springer Nature remains neutral with regard to jurisdictional claims in published maps and institutional affiliations.

## Supplementary Material

Supplementary Information

## Figures and Tables

**Table 1:** The putative components of TOR signaling pathway in cotton.

Protein name	Mammals	Yeast	*Arabidopsis*	*G. hirsutum*
SIN1	√	√		
RICTOR	√	√		
PI3Kα	√	√		
PI3Kβ	√	√		
PI3Kγ	√	√		
TSC1	√			
TSC2	√			
IRS	√			
SGK1	√	√		
Akt	√	√		
4EBP1	√	√	√	
AMPK	√	√	√	√
LKB1	√	√	√	√
mLST8	√	√	√	√
RAPTOR	√	√	√	√
mTOR	√	√	√	√
FKBP12	√	√	√	√
PDK1	√	√	√	√
S6K	√	√	√	√
RPS6	√	√	√	√
α4	√	√	√	√
E2F3	√	√	√	√
ATPBD3	√	√	√	√
TCTP	√	√	√	√

**Table 2:** Representative *G.hirsutum* differentially expressed genes related to JA signaling pathway.

*Arabidopsis* homologs	Gene ID	Log2(Fold change)	P-adjusted	Annotation
AOS	Gh_D06G0089	4.62	5.08E-04	allene oxide synthase
	Gh_D06G0087	2.41	5.08E-04	allene oxide synthase
	Gh_A06G0111	3.82	5.08E-04	allene oxide synthase
OPR3	Gh_D05G0339	1.23634	5.08E-04	oxophytodienoate-reductase 3
LOX	Gh_A08G1863	2.39364	5.08E-04	lipoxygenase 1
	Gh_D13G1129	1.50253	5.08E-04	lipoxygenase 1
	Gh_D08G2224	2.52251	5.08E-04	lipoxygenase 1
	Gh_A13G0888	1.44615	5.08E-04	lipoxygenase 1
ACX1	Gh_A11G2972	1.04194	5.00E-04	acyl-CoA oxidase 1
	Gh_A12G1964	0.861585	1.36E-03	acyl-CoA oxidase 1
	Gh_D11G0264	1.43708	5.08E-04	acyl-CoA oxidase 1
	Gh_D12G2143	0.951985	5.08E-04	acyl-CoA oxidase 1
JMT	Gh_A10G0863	2.33821	1.97E-02	jasmonate O-methyltransferase
	Gh_A12G0585	3.40333	5.80E-03	jasmonate O-methyltransferase
	Gh_A12G0584	2.53434	1.76E-03	jasmonate O-methyltransferase
CUL	Gh_D01G2280	-0.710028	1.16E-02	cullin 1
JAZ	Gh_A12G2441	1.31113	7.53E-03	jasmonate-zim-domain protein 3
	Gh_A06G0705	1.70817	1.93E-02	jasmonate-zim-domain protein 1
	Gh_A08G2199	2.22148	2.73E-02	jasmonate-zim-domain protein 1
	Gh_D08G2564	3.20572	1.88E-02	jasmonate-zim-domain protein 1
	Gh_D12G2567	0.745434	1.03E-02	jasmonate-zim-domain protein 3
TOPLESS	Gh_A08G1965	0.774929	6.36E-03	TOPLESS protein
	Gh_A05G1044	1.60034	5.08E-04	TOPLESS protein
MYC2	Gh_A09G2341	1.44412	4.52E-03	Transcription factor MYC2
