# Supplementary material for: The crosstalk between Target of Rapamycin (TOR) and Jasmonic Acid (JA) signaling existing in Arabidopsis and cotton
Source: Sci Rep. 2017 Apr 4;7:45830. doi: 10.1038/srep45830 (PMC5379187; doi:10.1038/srep45830)
Supplement: Supplementary Information [file srep45830-s1.pdf]

The crosstalk between Target of Rapamycin (TOR) and Jasmonic Acid (JA) signaling existing in *Arabidopsis* and cotton

Yun Song[1+], Ge Zhao[1+], Xueyan Zhang[1+], Linxuan Li[2], Fangjie Xiong[2], Fengping Zhuo[2], Chaojun Zhang[1], Zuoren Yang[1], Raju Datla[3], Maozhi Ren[2*], Fuguang Li[1*]

1 State Key Laboratory of Cotton Biology, Cotton Research Institute, Chinese Academy of Agricultural Sciences, Anyang, Henan Province, China
2 School of Life Sciences, Chongqing University, Chongqing, China
3 National Research Council of Canada, Saskatoon, Canada

[+] The authors contributed equally to this work.

[*] To whom correspondence should be addressed. Maozhi Ren: renmaozhi@cqu.edu.cn, Fuguang Li: aylifug@163.com.

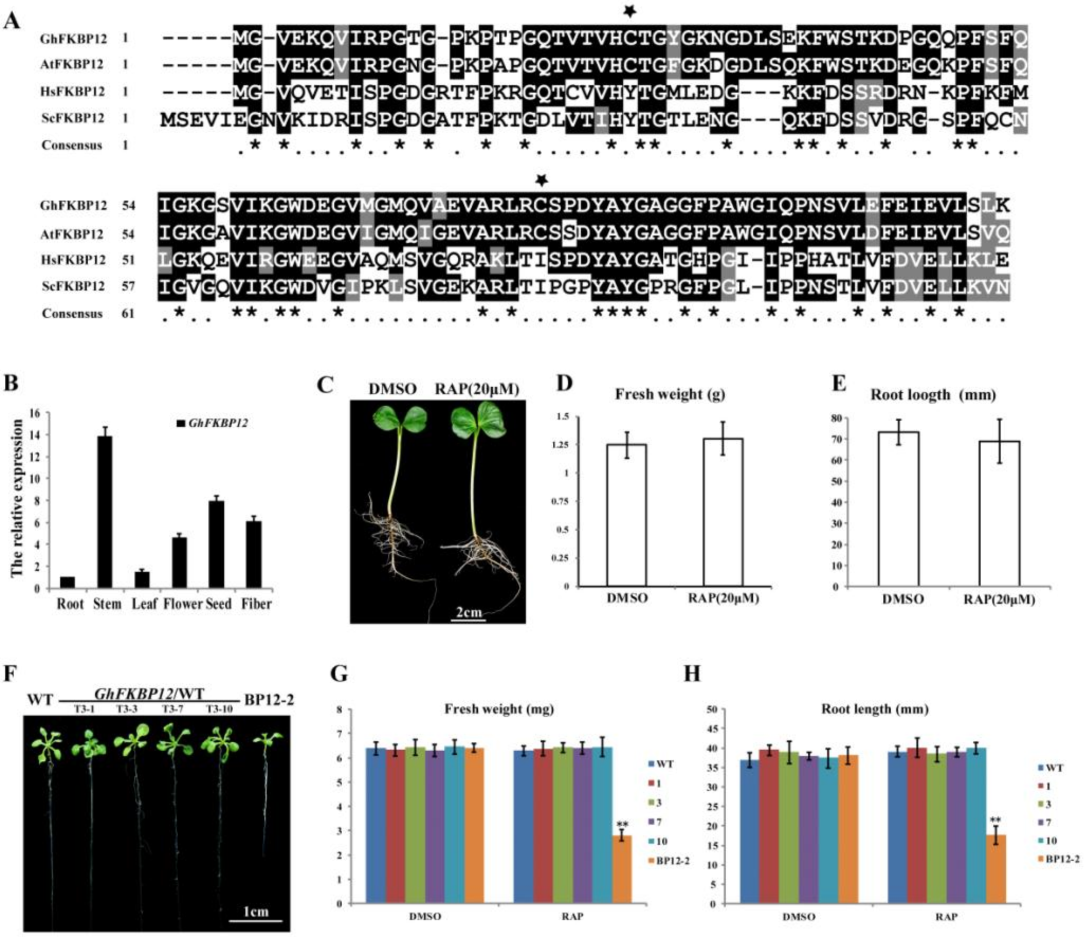

Supplementary Figure S1. Identification and characterization of *GhFKBP12*.
(A) The deduced amino acid sequence of *Gh*FKBP12 is compared with the sequences of FKBP12 from other representative organisms. Pentagrams above the sequences represent Cys^{26} and Cys^{80}. **(B)** The expression levels of *GhFKBP12* in the root, stem, leaf, flower, and fiber of cotton seedlings. **(C)** Rapamycin-insensitive phenotypes of cotton seedlings. Bar=2cm. Fresh weight **(D)** and Root length **(E)** of cotton seedlings exposed to RAP. **(F)** *GhFKBP12* overexpression transgenic *Arabidopsis* lines were insensitive to RAP treatment. Bar=2cm. Fresh weight **(G)** and Root length **(H)** of *GhFKBP12* overexpression transgenic *Arabidopsis* lines exposed to RAP. Error bars represent ±SE (n = 3). Each graph in (D), (E), (G) and (H) represents the average of 10 seedlings that were conducted in triplicate. Asterisks indicate significant differences between DMSO and indicated treatments at **$p < 0.01$ (Student's t-test). Three biological replicates were analyzed with similar results.

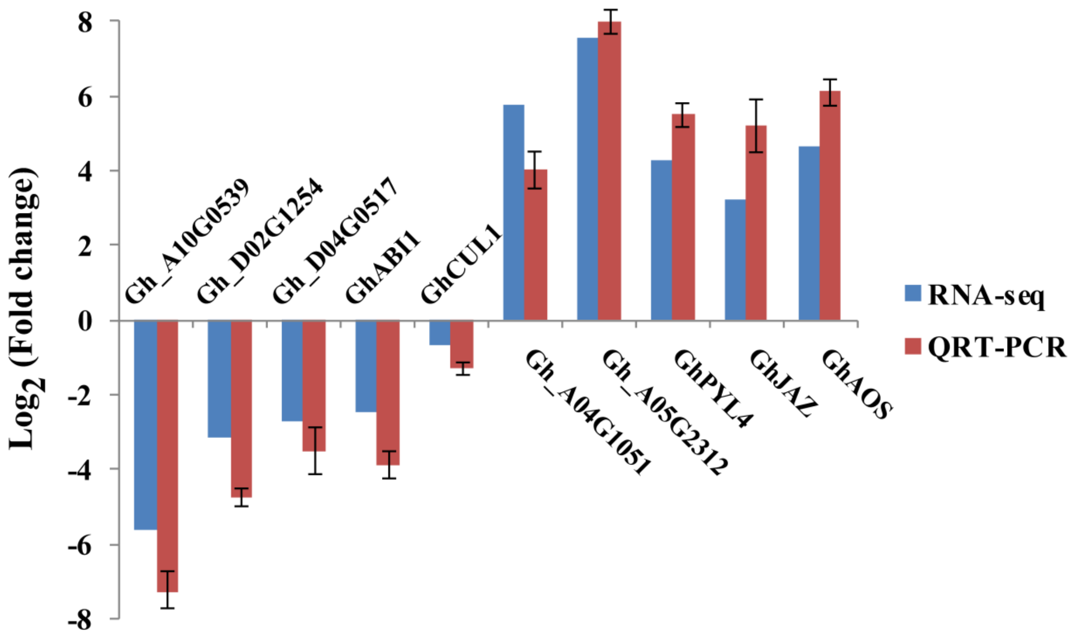

Supplementary Figure S2. Real-time PCR verification of the differentially expressed genes.

The results confirmed the down-regulation of the mRNA levels of Gh_A10G0539, Gh_D02G1254, Gh_D04G0517, GhABI1 (Gh_A13G1741), and GhCUL1 (Gh_D01G2280), and the up-regulation of the mRNA levels of Gh_D04G1051, Gh_A05G2312, GhPYL4 (Gh_D06G1764), GhJAZ (Gh_D08G2564) and GhAOS (Gh_D06G0089).

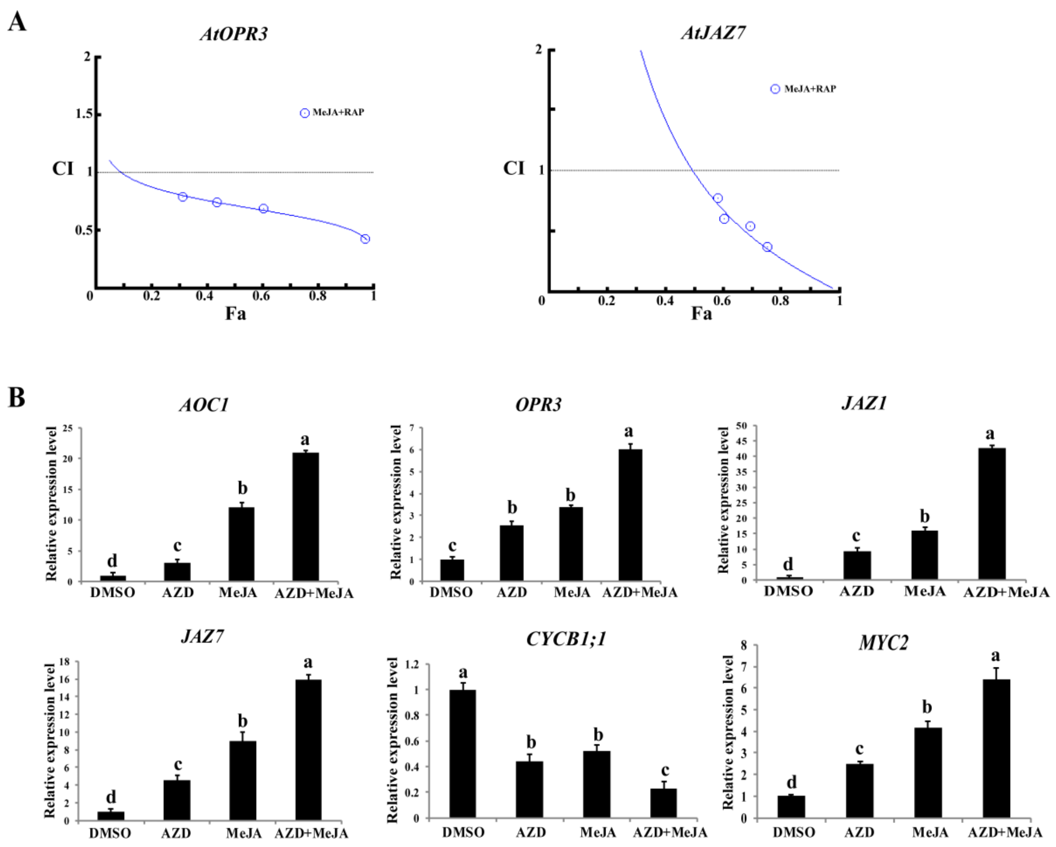

Supplementary Figure S3. AZD influences the expression levels of JA biosynthetic and responsive genes

(A) CI curves of *Arabidopsis* JA biosynthetic and responsive genes in BP12-2 seedlings. BP12-2 seedlings were first germinated on 1/2 MS medium for six days, and then transformed to plates containing DMSO, 10 μM MeJA, 0.35 μM RAP, and the combination of MeJA+RAP for 24 h. The expression levels of *AtOPR3* and *AtJAZ7* were analyzed in BP12-2 seedlings by QRT-PCR. The QRT-PCR data was used to obtain the CI curves in the CompuSyn software program. **(B)** The expression levels of JA biosynthetic and responsive genes in WT seedlings were influenced by MeJA and AZD. Expression levels of *AtAOC1*, *AtOPR3*, *AtJAZ1*, *AtJAZ7*, *AtCYCB1;1*, and *AtMYC2* were examined by QRT-PCR in WT seedlings following DMSO, 1 μM AZD, 10 μM MeJA, and 1 μM AZD+10 μM MeJA treatments. MS: Murashige & Skoog. Different letters indicate significant differences from the control.

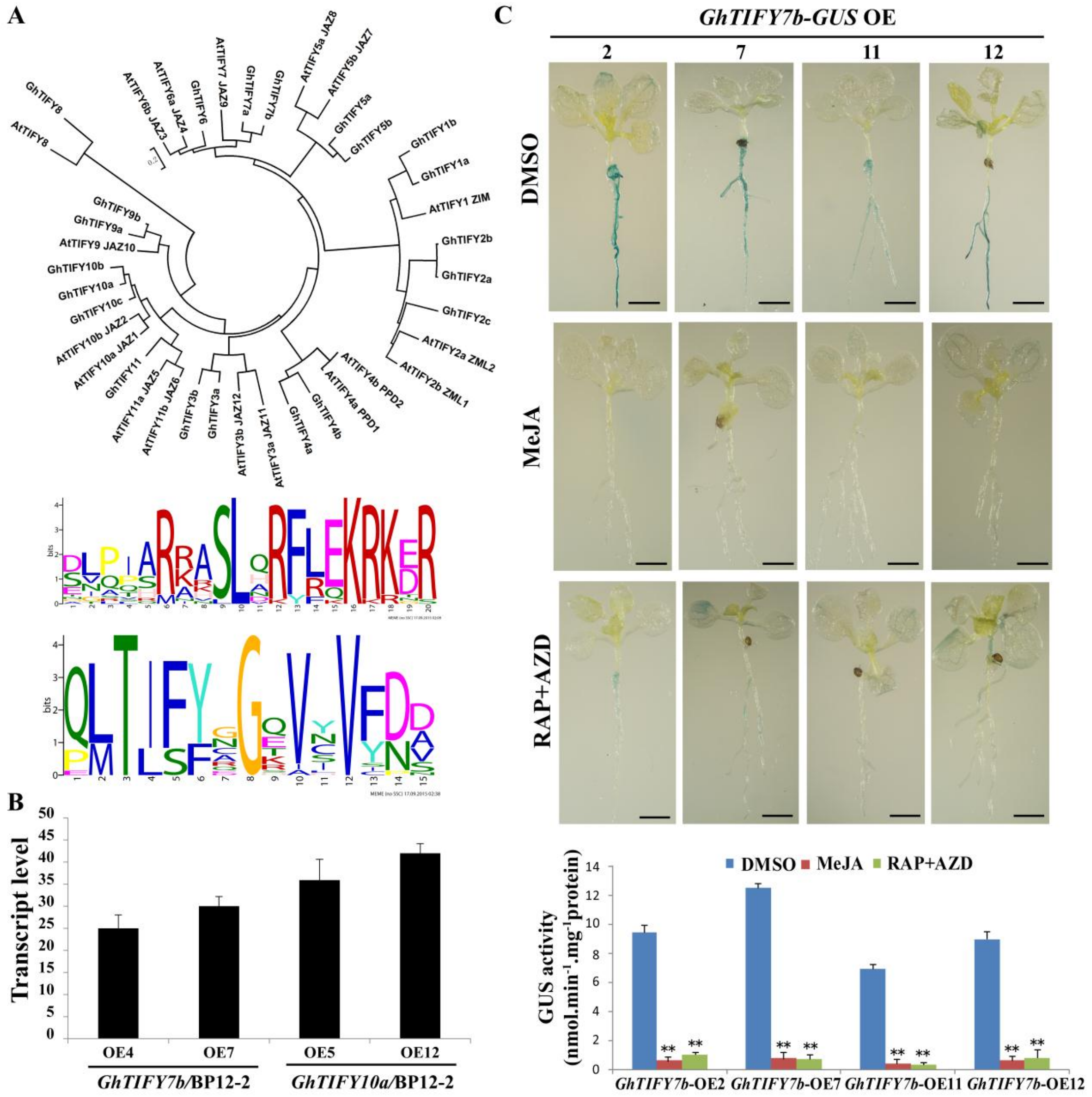

Supplementary Figure S4. TOR inhibition induces the degradation of GhTIFY7b.

(A) Phylogenetic tree construction and motif analysis of *G. hirsutum* TIFY family. 39 TIFY proteins from *G. hirsutum* and *Arabidopsis* were used to construct phylogenetic tree. The unrooted tree was constructed by MEGA4.0 using the neighbor-joining method. Motif analysis of *G. hirsutum* TIFY family was performed by using MEME [http://meme.sdsc.edu/meme (Bailey and Elkan, 1994)]. **(B)** QRT-PCR analysis of *GhTIFY7b* and *GhTIFY10a* transcripts from 10-day-old *GhTIFY7b* and *GhTIFY10a* overexpression transgenic lines. **(C)** *35S-GhTIFY7b-GUS* transgenic lines were exposed to DMSO, MeJA (10 μM) and the combination of RAP (0.35 μM) and AZD (1 μM) for 48 h. GUS activity of these transgenic lines was measured by fluorometric quantification. Error bars indicate ±SD of triplicates. T-test significant differences compared to that of DMSO (**P < 0.01).

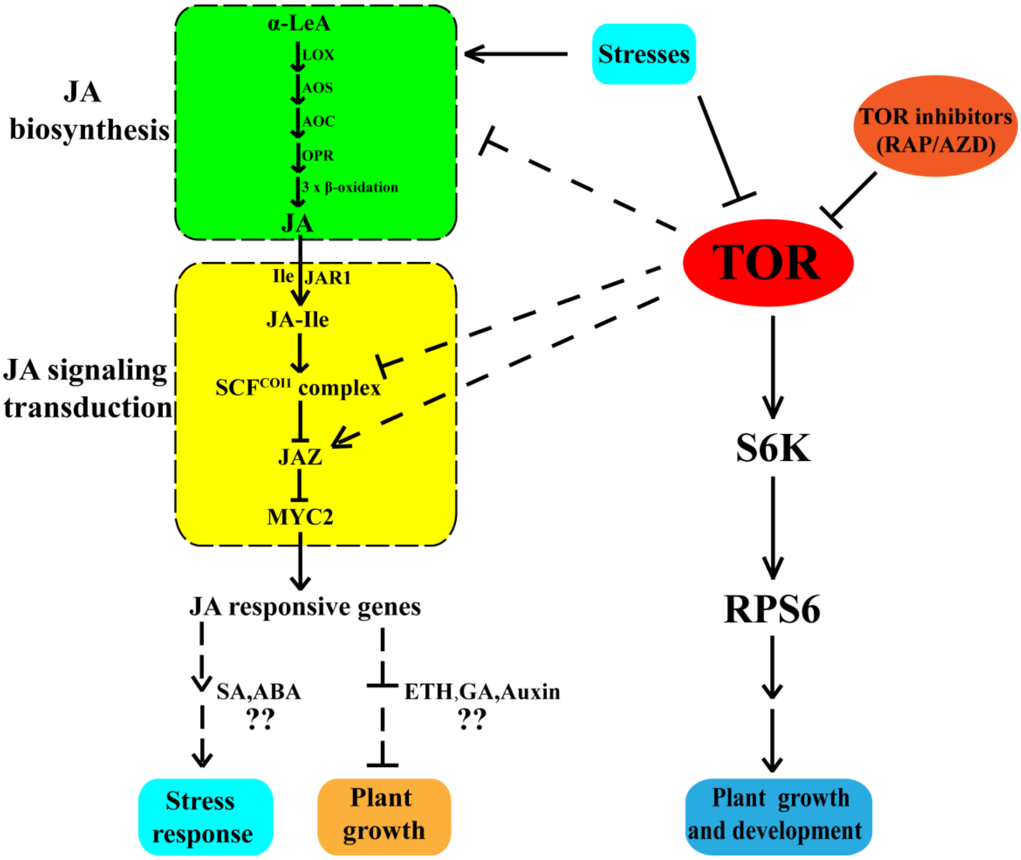

Supplementary Figure S5. The proposed model for the crosstalk between TOR and JA signaling pathway in plants.

TOR suppression represents a stress for plants. JA is an important regulator in plant stress responses. Thus TOR inhibition promotes JA synthesis, further reinforces JA signaling transduction, and finally modulates plant development and stress response. ETH: ethylene; GA: gibberellic acid; SA: salicylic acid; ABA: abscisic acid.

Supplementary Table S1. Cotton key elements of TOR signaling pathway

Protein name	Homo sapiens	Yeast	Arabidopsis	G. hirsutum	Chr location	Identity (%)
Target of rapamycin	mTOR	TOR1	TOR(AT1G50030)	TOR-like1(CotAD_08938)	1	77
		TOR2		TOR-like2(CotAD_50996)	12	78
Regulatory associate protein of TOR	mRAPTOR	KOG1	RAPTOR1(AT3G08850)	RAPTOR-like1(CotAD_14587)	1	73
			RAPTOR2(AT5G01770)	RAPTOR-like2(CotAD_54295)	11	71
				RAPTOR-like3(CotAD_76710)	11	77
				RAPTOR-like4(CotAD_33734)		74
Lethal with sec-13 protein 8	mLST8	LST8	LST8-1(AT3G18140)	LST8-like(CotAD_28305)	2	86
			LST8-2(AT2G22040)			
S6 kinase	mS6K	SCH9	S6K1 (AT3G08730)	S6K-like1(CotAD_27962)	11	67
			S6K2(AT3G08720)	S6K-like2(CotAD_57669)	11	67
				S6K-like3(CotAD_57170)	9	65
				S6K-like4(CotAD_16768)	9	64
				S6K-like5(CotAD_24426)	10	66
				S6K-like6(CotAD_02069)	3	56
Ribosome protein small subunit 6	mRPS6	RPS6A	RPS6A(AT4G31700)	RPS6-like1(CotAD_12177)	13	90
		RPS6B	RPS6B(AT5G01360)	RPS6-like2(CotAD_48139)	12	90
				RPS6-like3(CotAD_69695)	7	90
				RPS6-like4(CotAD_67556)	6	90
				RPS6-like5(CotAD_35661)	3	90
				RPS6-like6(CotAD_62620)	10	90
				RPS6-like7(CotAD_08552)	3	90
				RPS6-like8(CotAD_44061)	8	88
				RPS6-like9(CotAD_67343)	8	85
				RPS6-like10(CotAD_37696)	5	81
AMP activated protein kinase	mAMPK	SNF1	KIN10(AT3G01090)	AMPK-like1(CotAD_57320)	6	85
			KIN11(AT3G29160)	AMPK-like2(CotAD_52889)	6	85
				AMPK-like3(CotAD_24954)	7	84
Peutz-Jeghers syndrome protein	mLKB	LKB	CIPK12(AT4G18700)	LKB-like1(CotAD_22749)	7	79
				LKB-like12(CotAD_25726)	9	78
FK506-binding protein 12	mFKBP12	FPR1	FKBP12(AT5G64350)	FKBP12-like(CotAD_57498)	6	86
Type 2A-phosphatase-associated protein 46 Kd	α4	TAP42	TAP46(AT5G53000)	TAP46-like2(CotAD_08037)	9	74
E2Fa transcription factor	mE2F3	E2F3	E2Fa(AT2G36010)	E2Fa-like1(CotAD_69395)	6	47
				E2Fa-like2(CotAD_44637)	3	47
Translationally controlled tumor protein	mTCTP	TCTP	TCTP(AT3G16640)	TCTP-like1(CotAD_59347)	13	78
				TCTP-like2(CotAD_10920)	7	75
Repressor of LRR-extensin 1	mATPBD3	NCS6P	ROL5(AT2G44270)	ROL5-like1(CotAD_58361)	9	83
Phosphoinositidedependent protein kinase 1	mPDK1	PDK1	PDK1(AT5G04510)	PDK1-like1(CotAD_19462)	2	82
				PDK1-like2(CotAD_05560)	9	81
				PDK1-like3(CotAD_57445)	9	72
Phosphatase and tensin homologue	mPTEN	PTEN	PTEN(AT5G39400)	PTEN-like(CotAD_10161)	7	71
v-akt murine thymoma viral oncogene homolog	mAKT	AGO	/	/		
Tuberous sclerosis complex 1	mTSC1	/	/	/		
Tuberous sclerosis complex 2	mTSC2	/	/	/		
Insulin receptor substrate	mIRS	/	/	/		
Phosphatidylinositol 3-kinase α	mPI3Kα	VPS34	/	/		
Phosphatidylinositol 3-kinase β	mPI3Kβ	VPS34	/	/		
Phosphatidylinositol 3-kinase γ	mPI3Kγ	VPS34	/	/		
Rapamycin-insensitive companion of mTOR	mRICTOR	AVO3	/	/		

Supplementary Table S2. Summary of sequencing data quality.

Sample name	Raw reads	Clean reads	Error rate (%)	Q20 (%)	Q30 (%)	GC content (%)
DMSO-1	87648222	84899502	0.01	98.7	96.68	43.29
DMSO-2	83642900	80886794	0.01	98.7	96.68	44.52
DMSO-3	100769042	97549042	0.01	98.68	96.66	43.62
AZD-1	105265384	102555968	0.01	98.28	95.68	44.91
AZD-2	85311302	82552052	0.01	98.48	96.11	44.75
AZD-3	108585606	105774810	0.01	98.31	95.73	44.81

Supplementary Table S3. The auxin- and ABA- related DEGs under TOR inhibition in cotton seedlings.

Gene ID	Log2(Fold change)	P-adjusted	Annotation
Auxin signal transduction			
Gh_A01G0906	-1.11509	0.0033	SAUR-like auxin-responsive protein family
Gh_D08G1376	3.00217	0.0056	SAUR-like auxin-responsive protein family
Gh_D02G2218	4.98856	0.0026	SAUR-like auxin-responsive protein family
Gh_D09G1378	-1.93736	0.0105	SAUR-like auxin-responsive protein family
Gh_D08G1506	3.02994	0.0056	SAUR-like auxin-responsive protein family
Gh_A12G2619	2.47409	0.0041	SAUR-like auxin-responsive protein family
Gh_D06G0824	-1.64089	0.00065	SAUR-like auxin-responsive protein family
Gh_A04G1051	5.7355	0.002	SAUR-like auxin-responsive protein family
Gh_D04G1627	4.87043	0.00005	SAUR-like auxin-responsive protein family
Gh_D10G0502	4.9656	0.00105	SAUR-like auxin-responsive protein family
Gh_D09G0057	3.02815	0.0056	SAUR-like auxin-responsive protein family
Gh_D05G1269	1.26531	0.00175	Auxin-responsive protein IAA26
Gh_D05G0138	-1.6522	0.0019	Auxin-responsive protein IAA29
Gh_A05G0079	-1.32403	0.00565	Auxin-responsive protein IAA29
Gh_D05G3127	-1.52159	0.028495	Auxin-responsive protein IAA32
Gh_A07G1720	2.78226	0.00065	Auxin-responsive family protein
Gh_A11G1310	1.05421	0.00325	Auxin-responsive family protein
Gh_D10G1512	-1.47739	0.0055	AUX/IAA transcriptional regulator family protein
Gh_D08G0665	-1.24212	0.00795	AUX/IAA transcriptional regulator family protein
Gh_D09G2152	-2.21409	0.0016	AUX/IAA transcriptional regulator family protein
Gh_A11G2745	1.15699	0.0038	Auxin efflux carrier family protein
Gh_D11G3341	1.24854	0.00465	Auxin efflux carrier family protein
Gh_A02G1716	1.50872	0.00005	Auxin efflux carrier family protein
Gh_D03G0004	1.48094	0.00005	Auxin efflux carrier family protein
Gh_A12G2431	1.22712	0.00115	Auxin efflux carrier family protein
Gh_D07G0761	-1.14435	0.0004	Auxin efflux carrier family protein

Gh_D09G1548	-1.16548	0.0003	Auxin transporter-like protein 2
Gh_A01G1374	1.05761	0.00275	Auxin transporter-like protein 5
Gh_A12G0043	1.17039	0.00625	Endoplasmic reticulum auxin binding protein 1
Gh_D11G1989	1.34778	0.0008	Auxin-responsive GH3 family protein
Gh_A11G2575	2.16307	0.0038	Auxin-responsive GH3 family protein
Gh_D04G0261	1.36817	0.0077	Auxin-responsive GH3 family protein
Gh_A01G0547	1.82081	0.00005	Auxin-responsive GH3 family protein
Gh_A11G1993	2.07801	0.00025	Auxin-responsive GH3 family protein
Gh_A01G0546	1.07874	0.0056	Auxin-responsive GH3 family protein
Gh_D11G2945	2.28151	0.0017	Auxin-responsive GH3 family protein
Gh_A11G3061	-1.09312	0.0099	Auxin-responsive GH3 family protein
Gh_D11G0514	1.73646	0.00005	Auxin-responsive GH3 family protein
Gh_D01G0557	1.5714	0.00005	Auxin-responsive GH3 family protein
Gh_D01G0559	1.90798	0.00005	Auxin-responsive GH3 family protein
Gh_A08G0656	1.18067	0.000508	Auxin response factor 2
Gh_A06G2038	1.69827	0.04996	Auxin response factor 3
Gh_A10G1020	-1.23688	0.00452	AUX/IAA transcriptional regulator family protein
Gh_D08G0665	-1.24212	0.0378097	AUX/IAA transcriptional regulator family protein
Gh_D09G2152	-2.21409	0.0102869	AUX/IAA transcriptional regulator family protein
Gh_D10G1512	-1.47739	0.0280879	AUX/IAA transcriptional regulator family protein
Abscisic acid (ABA) signal transduction			
Gh_A03G1664	0.713625	0.0229998	ABRE binding factor 4
Gh_D02G2079	1.24754	0.0005082	ABRE binding factor 4
Gh_D05G2920	1.64544	0.0021294	Abscisic acid receptor PYL4
Gh_D06G1764	4.26558	0.0152996	Abscisic acid receptor PYL6
Gh_A06G1418	2.27885	0.001363	Abscisic acid receptor PYL6
Gh_D10G2388	1.73881	0.000508	Abscisic acid receptor PYL6
Gh_A10G2142	1.77238	0.0110832	Abscisic acid receptor PYL6
Gh_A03G0015	2.15838	0.001363	Abscisic acid receptor PYR1
Gh_D03G1860	1.53918	0.0157708	Abscisic acid receptor PYR1
Gh_A05G2234	-1.18954	0.002494	Abscisic acid responsive elements-binding factor 2
Gh_D08G1639	2.16181	0.000508	Abscisic acid 8'-hydroxylase 1
Gh_A08G1344	2.33311	0.000508	Abscisic acid 8'-hydroxylase 1
Gh_A03G1667	3.86987	0.000508	Abscisic acid 8'-hydroxylase 4
Gh_D02G2083	3.27764	0.000508	Abscisic acid 8'-hydroxylase 4
Gh_D09G0083	1.04533	0.00095	ABI5 binding protein 2
Gh_A13G1741	-2.49592	0.002129	Protein phosphatase 2C family protein ABI1
Gh_A12G2276	1.02912	0.000508	Protein phosphatase 2C family protein
Gh_D05G0451	3.22994	0.000508	Protein phosphatase 2C family protein
Gh_D05G0614	1.16139	0.0169583	Protein phosphatase 2C family protein
Gh_D02G1853	1.35169	0.0005082	Protein phosphatase 2C family protein
Gh_A10G1864	2.91615	0.0041967	Protein phosphatase 2C family protein
Gh_A04G0660	1.55443	0.0005082	Protein phosphatase 2C family protein
Gh_D02G0601	1.04686	0.0240786	Protein phosphatase 2C family protein

Gh_A11G2459	1.60285	0.0005082	Protein phosphatase 2C family protein
Gh_D11G2776	1.185	0.00095	Protein phosphatase 2C family protein

Supplementary Table S4. The GI50 values of MeJA, RAP and AZD alone or pairwise combination treatment in BP12-2.

	Single treatment(μM)			Combination treatment(μM)			
Exposure time(d)	MeJA	RAP	AZD	MeJA	RAP	AZD	CI
10	10	0.35	1	2	0.02		0.103
				2		0.05	0.251

Supplementary Table S5. Basic information of the *GhTIFY* family in cotton.

Gene name	Synonym	Group A ID	Group AD ID	Full CDS length(bp)
GhTIFY1a		Cotton_A_30531	CotAD_32387	867
GhTIFY1b		Cotton_A_24823	CotAD_68174	891
GhTIFY2a		Cotton_A_17214	CotAD_65649	939
GhTIFY2b		Cotton_A_15821	CotAD_35015	945
GhTIFY2c		Cotton_A_24824	CotAD_67271	1062
GhTIFY3a	*GhJAZ1*	Cotton_A_05658	CotAD_18763	687
GhTIFY3b	*GhJAZ2*	Cotton_A_14056	CotAD_40859	690
GhTIFY4a		Cotton_A_16991	CotAD_67677	1080
GhTIFY4b		Cotton_A_41299	CotAD_75535	1248
GhTIFY5a	*GhJAZ3*	Cotton_A_02904	CotAD_75527	363
GhTIFY5b	*GhJAZ4*	Cotton_A_12336	CotAD_27478	360
GhTIFY6	*GhJAZ5*	Cotton_A_01448	CotAD_21832	3345
GhTIFY7a	*GhJAZ6*	Cotton_A_36075	CotAD_41943	1095
GhTIFY7b	*GhJAZ7*	Cotton_A_36376	CotAD_67052	1092
GhTIFY8		Cotton_A_11516	CotAD_50802	1284
GhTIFY9a	*GhJAZ8*	Cotton_A_00049	CotAD_24822	732
GhTIFY9b	*GhJAZ9*	Cotton_A_10012	CotAD_02206	594
GhTIFY10a	*GhJAZ10*	Cotton_A_27840	CotAD_00351	723
GhTIFY10b	*GhJAZ11*	Cotton_A_11862	CotAD_22999	759
GhTIFY10c	*GhJAZ12*	Cotton_A_09418	CotAD_46116	786
GhTIFY11	*GhJAZ13*	Cotton_A_18896	CotAD_62298	813

Supplementary Table S6. Primers used in this study.

Experiment	Name	Sequence
Cloning	*Gh*FKBP12F	GCGGCCGCATGGGAGTAGAGAAGCAAGTC
Cloning	*Gh*FKBP12R	CCTGCAGGCTTCAAGCTTAGGACTTCAAT
Cloning	*At*COI1F	GCGGCCGCATGGAGGATCCTGATATCAAG
Cloning	*At*COI1R	CCTGCAGGTATTGGCTCCTTCAGGACTCT
Cloning	*Gh*TIFY1aF	GCGGCCGCATGTACGGACAGTCTCAGCCCATG
Cloning	*Gh*TIFY1aR	CCTGCAGGGTTCTCGGCAATTAGAGCCGAG
Cloning	*Gh*TIFY1bF	GCGGCCGCATGTACGGACAATCTCAGCCTATG
Cloning	*Gh*TIFY1bR	CCTGCAGGGTGCTCGGCAATGCCGATTAGAG
Cloning	*Gh*TIFY2aF	GCGGCCGCATGGCGAATTCGAATCTCCACTC
Cloning	*Gh*TIFY2aR	CCTGCAGGTCTTTCGGAAGTCACACCAGAG
Cloning	*Gh*TIFY2bF	GCGGCCGCATGGCGAATTCGAATCACCAGC
Cloning	*Gh*TIFY2bR	CCTGCAGGTCTTTCAGCAGTAACAGTGGAG
Cloning	*Gh*TIFY2cF	GCGGCCGCATGGCGGCTGCGAATCCACGGC
Cloning	*Gh*TIFY2cR	CCTGCAGGATCAAAACTAGGAGGAATGTCG
Cloning	*Gh*TIFY3aF	GCGGCCGCATGGAAGAAGAGGCTGAATCACGC
Cloning	*Gh*TIFY3aR	CCTGCAGGGGCAACATGAGCTGGAGCTTTTGG
Cloning	*Gh*TIFY3bF	GCGGCCGCATGGAAGGGGAAGCTGGTTCATAC
Cloning	*Gh*TIFY3bR	CCTGCAGGTGCAACATGAGCTGGAGCTTTTGG
Cloning	*Gh*TIFY4aF	GCGGCCGCATGTCAACGGGAGAAATGGTTTC
Cloning	*Gh*TIFY4aR	GCGGCCGCATGTCAACGGGAGAAATGGTTTC
Cloning	*Gh*TIFY4bF	GCGGCCGCATGGAGGCTGGGGTAACGACGAC
Cloning	*Gh*TIFY4bR	CCTGCAGGAAGGCCCTCTCCAGTTACTTTTTCC
Cloning	*Gh*TIFY5aF	GCGGCCGCATGAGACGAAACTGCAACTTGG
Cloning	*Gh*TIFY5aR	CCTGCAGGATAGTAAGGGGAGGTAGCTTGG
Cloning	*Gh*TIFY5bF	GCGGCCGCATGAGACGAAACTGCAACTTAG
Cloning	*Gh*TIFY5bR	CCTGCAGGATGGTAAGGAGAGGTAGCTTGG
Cloning	*Gh*TIFY6F	GCGGCCGCATGGAGGATCCTGATATCAAG
Cloning	*Gh*TIFY6R	CCTGCAGGATTGATGGCTTGTAAAGGACTA
Cloning	*Gh*TIFY7aF	GCGGCCGCATGGAGAGAGATTTTCTGGGTTTG
Cloning	*Gh*TIFY7aR	CCTGCAGGTGCATTTGATCCCTGGGTTGTAC
Cloning	*Gh*TIFY7bF	GCGGCCGCATGGAGAGAGATTTTCTGGGTTTG
Cloning	*Gh*TIFY7bR	CCTGCAGGTGCATTCGATTCCATGGTAGTTG
Cloning	*Gh*TIFY8F	GCGGCCGCATGGCTGCTGTTCTGAAAATGG
Cloning	*Gh*TIFY8R	CCTGCAGGTACCTCTCTTTTATCTTCAGTACC
Cloning	*Gh*TIFY9aF	GCGGCCGCATGTCCAGAGCTACCGTCGAGC
Cloning	*Gh*TIFY9aR	CCTGCAGGGCCGTTGATGGGTGACCGTGCG
Cloning	*Gh*TIFY9bF	GCGGCCGCATGTCGAGAGCTAGCGTCGAGCTT
Cloning	*Gh*TIFY9bR	CCTGCAGGCCAAGCATATGGAGATGCACAAG
Cloning	*Gh*TIFY10aF	GCGGCCGCATGTCGTCTTGCTCGGAATCTAC
Cloning	*Gh*TIFY10aR	CCTGCAGGTGGTGATTGAGCAGCCAAACCG
Cloning	*Gh*TIFY10bF	GCGGCCGCATGTTTGGTTCACCGGAATATAC
Cloning	*Gh*TIFY10bR	CCTGCAGGCTGTAGTGATTCAACAGCTAAACC

Cloning	*Gh*TIFY10cF	GCGGCCGCATGTCGTGTTCACCGGAATTTTTGG
Cloning	*Gh*TIFY10cR	CCTGCAGGCGGAGATTGAGCAGCCAAACCG
Cloning	*Gh*TIFY11F	GCGGCCGCATGTCTAATTTAGGGCAAAAATC
Cloning	*Gh*TIFY11R	CCTGCAGGTAAGTTGAGATCAATGTCTCTTG
Realtime PCR	*At*AOC1F	CTCCGAGACCAAACCTAAAGC
Realtime PCR	*At*AOC1R	CTCCGAGACCAAACCTAAAGC
Realtime PCR	*At*OPR3F	TGTAGTTTCAGCCATAGGAGC
Realtime PCR	*At*OPR3R	TAGTGGGTCAGAATCAGTTGC
Realtime PCR	*At*JAZ1F	GAGCAAAGGCACCGCTAATA
Realtime PCR	*At*JAZ1R	TGCGATAGTAGCGATGTTGC
Realtime PCR	*At*JAZ7F	ATCCCAAACAATTCGACTCG
Realtime PCR	*At*JAZ7R	GGAAGTTGCTTGAATCCGAA
Realtime PCR	*At*CYCB1;1F	CCGGAACTGAATCTGCTTAGG
Realtime PCR	*At*CYCB1;1R	GCGACTCATTAGACTTGTTCA
Realtime PCR	*At*MYC2F	GAACGAAGATAAAGTTCTATCA
Realtime PCR	*At*MYC2R	CAACCGCTCGTAACGCGTAGA
Realtime PCR	*At*ACTIN2F	TTGACTACGAGCAGGAGATGG
Realtime PCR	*At*ACTIN2R	ACAAACGAGGGCTGGAACAAG
Realtime PCR	*Gh*FKBP12F	GTCAGACCGTCACCGTTCACT
Realtime PCR	*Gh*FKBP12R	ACACCTTCATCCCATCCTTTT
Realtime PCR	*Gh*TOR1F	TTCTTCAGGCTGTCAATCAAC
Realtime PCR	*Gh*TOR1R	TGTTCCACTTCTCGGTTATCTC
Realtime PCR	*Gh*TOR2F	TCTTGCTCCTCAATACCGACAG
Realtime PCR	*Gh*TOR2R	AAAGGGACACCACCCAACATAG
Realtime PCR	*Gh*TIFY7bF	TCCCACCCTTGGCACTGTT
Realtime PCR	*Gh*TIFY7bR	AGTCAATTGAGCAGGTGATCCA
Realtime PCR	*Gh*TIFY10aF	CGTCTTGCTCGGAATCTA
Realtime PCR	*Gh*TIFY10aR	AATCACTTGTCCACCATAAA
Realtime PCR	*Gh*Histone3F	CCTTGTGGGTCTTTTTGAA
Realtime PCR	*Gh*Histone3R	AACTGGATGTCCTTGGGC
Realtime PCR	Gh_A10G0539F	TCCCTGCCATTAAAGCAGCTC
Realtime PCR	Gh_A10G0539R	TCCAAGGTGCTCCAGAATTGTT
Realtime PCR	Gh_D02G1254F	CCGGCTGGTTGTTCCCTATT
Realtime PCR	Gh_D02G1254R	CATGGCAATCCGAGGAGACA
Realtime PCR	Gh_D04G0517F	ACTCTAGCACTCAAGGGTGG
Realtime PCR	Gh_D04G0517R	GAGAGGGGCGAAGAAAGCTG
Realtime PCR	GhABI1F	CACTTCGGTGATCGGGAGAC
Realtime PCR	GhABI1R	ACGTGACCACACGTGAGAGA
Realtime PCR	GhCUL1F	GAAAAACGGGACGTGGTTGG
Realtime PCR	GhCUL1R	TCAAACCTTGAAACAGTCTAATGCT
Realtime PCR	Gh_D04G1051F	CCATTTCGCGGTTTACGTCG
Realtime PCR	Gh_D04G1051R	CCCCTCACGGGATAATCGAA
Realtime PCR	Gh_A05G2312F	TGCAATATCTGATCGGCGCT
Realtime PCR	Gh_A05G2312R	GCTCGCTCTTGGAATGAGGT

Realtime PCR	GhPYL4F	GACCTTTTTCCCTCTTCCTACTG
Realtime PCR	GhPYL4R	GGAGCAACACTGGTTAGGAC
Realtime PCR	GhJAZF	GAACGAATCCGGCCTAGGAG
Realtime PCR	GhJAZR	TCGAGCAATGGGTAGATCGC
Realtime PCR	GhAOSF	ATGGATCGGTAATGCCGGAC
Realtime PCR	GhAOSR	CCACCGATTTCATCAACGGC
